# Hypoglycaemia after Initiation of CFTR Modulator Therapy in a Cystic Fibrosis Patient without Diabetes

**DOI:** 10.1155/2023/9769119

**Published:** 2023-12-23

**Authors:** Marie Yskout, Joke Vliebergh, Hakan Bor, Lieven Dupont, Natalie Lorent, Pascal Van Bleyenbergh, Pieter Gillard, Bart Van der Schueren, Ann Mertens, Chantal Mathieu, Roman Vangoitsenhoven

**Affiliations:** ^1^UZ Leuven, Endocrinology, Leuven, Belgium; ^2^Nutrition and Dietetic, Gumushane University, Gumushane, Türkiye; ^3^Chronic Diseases and Metabolism, KU Leuven, Leuven, Belgium; ^4^Pneumology, UZ Leuven, Leuven, Belgium

## Abstract

**Introduction:**

Cystic fibrosis transmembrane regulator (CFTR) modulator therapies improve respiratory function and glycaemic control in patients with cystic fibrosis (CF). The direct effect of CFTR modulator therapies on pancreatic function in patients without preexisting diabetes remains unclear. *Case Presentation*. An 18-year-old female with CF caused by F508del/F508del mutation, who had no diabetes, developed postprandial hypoglycaemias 6 months after initiation of elexacaftor, tezacaftor, and ivacaftor combination therapy (ETI). Symptoms were persisted after brief discontinuation of ETI, but her symptoms and time-in-hypoglycaemia had improved remarkably by avoiding high glycaemic index-foods. *Discussion*. This case of hypoglycaemia associated with CFTR modulator therapy in a patient without preexisting diabetes suggests that CFTR modulator therapy has the potential to directly affect glucose homeostasis. There might be an improvement in insulin secretion as well as a reduction in systemic insulin resistance.

**Conclusion:**

Treatment of CF patients without diabetes with CFTR modulator therapies can cause recurrent hypoglycaemic episodes which resolve with dietary measures.

## 1. Introduction

Cystic fibrosis (CF) is a genetic disorder in which a dysfunctional cystic fibrosis transmembrane regulator (CFTR) chloride channel results in multimorbidity, including severe respiratory disease and reduced pancreatic endocrine and exocrine functions [1, 2]. Cystic fibrosis-related pancreatic endocrine dysfunction is known to result in failure of the glucose homeostasis causing fasting- and postprandial hypoglycaemia's, impaired glucose tolerance and/or cystic fibrosis-related diabetes (CFRD) [3, 4]. CFTR modulator therapies have revolutionised the treatment of patients with CF by improving respiratory function. Recently, the more potent CFTR modulator therapies have additionally shown the ability to lower glucose levels in patients with CF-associated diabetes [5–7]. However, the effect of CFTR modulator therapy on pancreatic function in patients without preexisting diabetes remains unclear. Patients with CF without diabetes are often taught to have a high intake of carbohydrates in an attempt to avoid weight loss and steatorrhea. As insulin secretion and insulin resistance could improve with initiation of CFTR modulator therapy, this may lead to hypoglycaemia in patients with a high intake of fast-acting carbohydrates [8, 9].

## 2. Case Presentation

**Figure 1:**
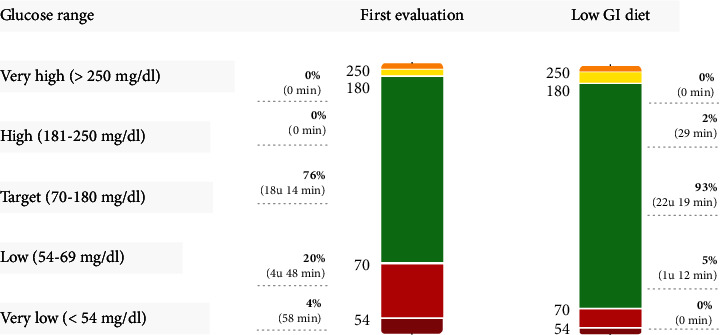
Time in range results from intermittent scanning and continuous glucose monitoring after the start of the CFTR modulator therapy (left bar) and after adoption of a low glycaemic index (GI) diet (right bar). The CGM data are summarized as the average of the previous 14 days and categorized as time-in-range based on consensus recommendations. Note that the time spent in “low” and “very low” decreased with the low GI diet, in favour of increase time “in range.”

Here, we report the case of an 18-year-old female with CF based on F508del/F508del genotype who was admitted to the hospital because of an increasing number of episodes of nausea, weakness, dizziness, trembling, and tinnitus. On questioning, it has been found that the symptoms exclusively occurred approximately 1 hour after the ingestion of food and resolved quickly after the ingestion of rapid sugars. The patient's history showed that her CF was complicated with bronchiectasis, exocrine pancreatic dysfunction, and nasal polyposis which were treated with a combination therapy of preventative antibiotics, inhalation therapy, vitamins, pancreatic enzymes, and a high-calorie diet. Six months prior to the current admission, the patient was started on the CFTR modulator therapy combination of 2 CFTR correctors, elexacaftor and tezacaftor, and 1 potentiator, ivacaftor (ETI), via a compassionate use programme. The therapy had a clear beneficial effect on her respiratory symptoms, and she had initially gained 5 kilograms. The episodic complaints had started 2 months prior to the current hospital admission and had obtained more and more frequent over time, and by the time of admission, she experienced frequent daily episodes which greatly impaired her daily life and food intake. She did not report an association between her postprandial symptoms and her level of physical activity. A brief interruption of ETI for 5 days had not resolved the postprandial symptoms, and at the time of admission, her weight had returned to her pretherapy weight with a body mass index of 20.8 kg/m^2^. Based on these findings, the patient was given a glucose sensor, and finger pricks were performed at the time of symptoms; results showed frequent glycaemic values below 60 mg/dl. Further biochemical testing during an episode of postprandial nausea confirmed a low venous blood glucose level (63 mg/dl) in combination with an unadjusted high level of insulin (141 pmol/l) and c-peptide (1.27 nmol/l). An intermittently scanning continuous glucose monitor showed frequent occurrence of postprandial reactive hypoglycaemia with 20% of the time spent in “low” (54–69 mg/dl) and 4% in “very low” glycaemic range (<54 mg/dl) (Figure 1). Further clinical and biochemical evaluation excluded underlying infection or other comorbidities. Revision of her medical file showed no history of hyperglycaemia (no previous oral glucose tolerance tests available; maximal postprandial peak glucose reading 179 mg/dl), and she had never used exogenous insulin, nor any other type of glucose lowering agent. However, the glycated haemoglobin level (HbA1c) had dropped from 6.2% to 5.4% after the initiation of ETI.

The treatment with ETI was, as was requested by the patient, continued at the same dose. The patient was advised to specifically limit her intake of carbohydrates with a high glycaemic index without changing her overall carbohydrate intake. Over the course of the next 3 months, her symptoms improved notably in frequency and severity. This clinical improvement was reflected in the continuous glucose monitor reading, showing a clear reduction in time spent in “low” (5% of time) and “very low” glycaemic range (0% of time) (Figure 1 panel B).

## 3. Discussion

Endocrine pancreatic dysfunction is a frequent complication seen in patients with CF and can result in the development of CFRD. Its pathophysiology is believed to be multifactorial with (1) a decrease in insulin secretion by the pancreatic beta cells, either direct or indirect, and (2) a rise in insulin resistance [8, 10]. Even though the exact role of CFTR in the pathophysiology of endocrine pancreatic dysfunction is unclear, this case report supports the preliminary notion that CFTR modulator therapy has the potential to affect glucose homeostasis in patients with CF without prior CFRD. In recent years, as CFTR modulator therapies have become available for widespread use, multiple small studies have suggested a link between CFTR and glucose homeostasis.

A retrospective study reported a (near) resolution of CFRD in 4 out of 14 patients who were treated with ivacaftor monotherapy or combination therapy with ivacaftor/lumacaftor [7]. Positive effects on glucose homeostasis have also been reported in patients without preexisting CFRD after the initiation of ivacaftor. An open label small pilot study conducted in 5 CF patients with the G551D mutation, of which 3 patients without CFRD, showed that the insulin response to oral glucose increased by 66–178% in all but one patient after 4 weeks of therapy with ivacaftor [11]. This study also showed that insulin secretion itself improved after glucose stimulation and that the improvement was greatest in the patient who had had impaired glucose tolerance preinitiation of therapy. In 2016, an improvement in insulin secretion was reported 16 weeks after initiation of ivacaftor in one patient with impaired insulin secretion pretherapy initiation [12]. However, the findings are inconsistent, especially in patients receiving lumacaftor/ivacaftor combination therapy [13, 14].

Since ETI was approved for clinical practice, multiple retrospective studies have reported the effect of ETI initiation on glucose regulation in CF patients with and/or without CFRD; unfortunately, no prospective studies have been published so far [15–17]. A recent systematic review found a seemingly a more consistent beneficial effect on blood glucose after the initiation of ETI, with eight out of eleven studies, with a patient sample size greater than one, showing a statistically significant improvement in at least one blood glucose measure in both patient groups [16]. Two studies reported separately the effects in CF patients without CFRD. In the study of Scully et al., the effect of ETI initiation was studied in 34 adults with CF, and 9 of them had no associated CFRD [15]. This study showed improvements in average and peak sensor glucose as well as % time in target range 70–180 mg/dL, and the effects were largest in patients with preexisting CFRD. In contrast, the study of Petersen et al. found a significant reduction in HbA1c after the initiation of ETI in 57 patients without CFRD of −0.16 (*p* < 0.005) as opposed to a reduction of −0.17 (*p*=0.25) in the 46 patients with CFRD [17]. None of these studies have shown a significant effect on hypoglycaemia, neither reported nor measured.

The possible underlying mechanism remains unclear; one could argue that insulin resistance or beta-cell function or both might be affected. Indeed, a relief of chronic inflammation, especially in the respiratory tract and lungs, might yield reduced insulin resistance and thus improved glucose homeostasis. However, it is also possible that an indirect effect through reduction of pancreatic inflammation, glucagon secreting alpha cells, or indeed other neurohormonal factors affects the beta cells insulin secretory capacity causing an imbalance. Two studies investigating the effect of CFTR on beta cells and pancreas function suggested that CFTR has an important impact on pancreas function and pancreatic beta-cell function and thus insulin secretion [8, 9]. This could suggest that patients with sufficient remaining beta-cell function would be most susceptible to the hypoglycaemic side effect after initiation of ETI.

In our clinical case report, neuroglycopenic symptoms were present postprandially, resembling the known phenomenon of reactive hypoglycaemia in patients who have exaggerated insulin secretion in response to fast-acting carbohydrates. This was clearly shown in the results of the continuous glucose monitor. Hypoglycaemic episodes have been reported previously in patients using CFTR modulator therapy while on insulin therapy, sometimes with continuing hypoglycaemia events after all insulin therapy was stopped (5). However, the current case is, to the best of our knowledge, the first case of clinically relevant hypoglycaemia in a patient without overt CFRD and previous insulin use. Together with a clinically significant decrease in HbA1c level, these observations suggest that there is an improvement in insulin secretion, either temporary or permanent, and not only reduction of insulin resistance.

## 4. Conclusion

This case report describes recurrent hypoglycaemic episodes and improved HbA1c levels after initiation of CFTR modulator therapy, even in the absence of prior CFRD. Physicians should be aware of this possible side effect before initiating CFTR modulator therapy and should provide proper nutritional counselling to patients receiving CFTR modulator therapy. Finally, larger and more long-term longitudinal studies are necessary to better understand the underlying mechanisms and the implications they bring to improve and maybe also prevent CFRD.

## Data Availability

The data used to support the findings of the study are available from the corresponding author upon reasonable request.
